# Female Reproductive Performance and Maternal Birth Month: A Comprehensive Meta-Analysis Exploring Multiple Seasonal Mechanisms

**DOI:** 10.1038/s41598-019-57377-9

**Published:** 2020-01-17

**Authors:** Mary Regina Boland, Martin Fieder, Luis H. John, Peter R. Rijnbeek, Susanne Huber

**Affiliations:** 1 Department of Biostatistics, Epidemiology and Informatics, Perelman School of Medicine, University of Pennsylvania, Philadelphia, USA; 2 Institute for Biomedical Informatics, University of Pennsylvania, Philadelphia, USA; 3 Center for Excellence in Environmental Toxicology, University of Pennsylvania, Philadelphia, USA; 4 Department of Biomedical and Health Informatics, Children’s Hospital of Philadelphia, Philadelphia, USA; 5 Department of Evolutionary Anthropology, University Vienna, Vienna, Austria; 6 Department of Medical Informatics, Erasmus University Medical Center, Rotterdam, The Netherlands

**Keywords:** Epidemiology, Epidemiology, Outcomes research

## Abstract

Globally, maternal birth season affects fertility later in life. The purpose of this systematic literature review is to comprehensively investigate the birth season and female fertility relationship. Using PubMed, we identified a set of 282 relevant fertility/birth season papers published between 1972 and 2018. We screened all 282 studies and removed 131 non-mammalian species studies on fertility and 122 studies that were on non-human mammals. Our meta-analysis focused on the remaining 29 human studies, including twelve human datasets from around the world (USA, Europe, Asia). The main outcome was change in female fertility as observed by maternal birth month and whether this change was correlated with either temperature or rainfall. We found that temperature was either strongly correlated or anti-correlated in studies, indicating that *another* factor closely tied to temperature may be the culprit exposure. We found that rainfall only *increases* fertility in higher altitude locations (New Zealand, Romania, and Northern Vietnam). This suggests the possibility of a combined or multi-factorial mechanism underlying the female fertility – birth season relationship. We discuss other environmental and sociological factors on the birth season – female fertility relationship. Future research should focus on the role of birth season and female fertility adjusting for additional factors that modulate female fertility as discussed in this comprehensive review.

## Introduction

Over 12% of reproductive age women (15–44 years) in the United States of America suffer from infertility or impaired fecundity^1^, with some estimates as high as 16%^2^. European countries show similarly high infertility rates with Great Britain at 17%^3^. In developing countries, the rate of infertility is estimated at 1 in 4 couples^4,5^. In addition to rising infertility rates among humans, increases in infertility and reproductive disorders were observed in dairy cows^6^. Dairy researchers found that a cool, low-temperature, environment appeared to preserve fertility and reduce the risk of reproductive disorders^6^ furthering support for a relationship between temperature and female fertility. Although at least one study points to other factors besides temperature that play a role more significant role at the country-level on female fertility^7^.

Maternal birth season effects female fertility outcomes^8^, including in both hunter/gather societies (e.g., Hiwi of Venezuela)^9^ and industrialized societies^8,10^. Birth season can affect later risk of disease through alterations in the prenatal environment that alter growth patterns and processes^11^. In addition, the known relationship between temperature and fertility^6^ could also manifest itself in a birth season relationship.

The main biological mechanism underlying a birth season relationship with female fertility remains the hypothesis that oocytes’ exposure to high temperatures at birth and shortly thereafter results in increased oocyte loss early in life^10,12–14^. Importantly, the female reproductive system, unlike males, is established early with females being born with their lifetime maximum number of oocytes^15–17^. Oocyte count is thought to be linked to fertility^18^. Therefore, researchers posit that this initial reduction in oocyte volume at the time of the woman’s birth thereby reduces a woman’s fertility when she goes to bear children of her own later in life. In addition, there are many studies among cows kept for dairy purposes, demonstrating the important role of temperature in female conception rates^19–21^.

This systematic review will comprehensively explore the literature underlying the maternal birth season – impaired female fertility paradigm while drawing from research conducted in humans. We will also discuss hypotheses supported by the literature and corresponding biological mechanisms to understand the effects observed in the literature and to place these findings in broader scientific context.

## Methods

This project was conducted using PRISMA guidelines^22^.

### Systematic review of literature

Our literature review focuses on fertility effects resulting from birth seasonal mechanisms. Therefore, we searched for relevant literature articles in PubMed as it is a freely and publically accessible database. All research funded by the National Institutes of Health within the United States of America are required to be deposited within PubMed. We used PubMed Central, an open access subset of PubMed, to obtain the full text of articles when possible. We also used site licenses from the University of Pennsylvania libraries to obtain the full texts of manuscripts retrieved via our initial query from PubMed. The following search query was used to identify relevant fertility/birth season papers:

“("fertility" OR "reproductive") AND ("birth season" OR "birth month" OR "high stress season")”.

We decided on the query detailed above after iteratively refining our query based on preliminary review of the search results obtained. The query interface within PubMed automatically maps the terms to their respective Medical Subject Headings (MESH) terms using PubMed Automatic Term Mapping^23^. After querying PubMed using the query detailed above, we then manually reviewed all retrieved abstracts and titles to identify the species studied in the papers. In addition to the species information and species type (e.g., primate, mammal), we also captured the location where the study was conducted (which can differ from the affiliations of the researchers), the time-scale of seasonal information captured (e.g., 12-month season, 4-month season, 2-month season), whether the study was a laboratory study, field research or epidemiological study, and whether the full-text of the paper was available and whether the data was readily available. These details were annotated by two personnel (MRB and a work study assistant) for all studies retrieved using the query above by two personnel.

We then chose to focus on human-only studies and to exclude studies involving non-human mammals and non-mammals (e.g., plants, fish). If the full-text of the manuscript was unavailable after multiple avenues had been explored (e.g., JSTOR, requesting manuscript from authors) then the manuscript was not included in our review. We also excluded studies where birth month was used as a control variable because in those studies the relationship between birth month/season and fertility was not being studied explicitly (although the effect was being removed via a covariate in a model).

### Selection of studies for inclusion in meta-analysis

We investigated all studies involving human data as these were the focus on this research. We also gleaned some helpful insights from the non-human mammal studies where appropriate. However, we used very specific criteria for a study to be included in our meta-analysis. First the study had to involve human data (we excluded all non-human mammals, primates, and other species). Second, the study had to involve a seasonal investigation on a 12-month time scale (therefore we excluded studies using 2-month and 4-month seasonal windows). Seasonal studies on a 2-month season (e.g., monsoon vs. dry season) are not readily translatable to a 12-month timescale without making a series of assumptions and therefore these were excluded from the meta-analysis. Third the study had to have data readily accessible to our research team. This could be in the form of a figure detailing the rates by 12-month season, or in the form of a table or downloadable.csv or.txt file that contained information at the 12-month timescale.

### Meta-Analysis of studies for birth season – fertility effect and environmental exposures

Meta-analysis was used to determine whether or not the birth season results reported demonstrated an overall correlation with temperature across all datasets included. The DerSimonian-Laird (DSL) random-effect meta-analytical method^24^ was used to compute the correlation between the exposure (e.g., temperature, rainfall) and female fertility. The DSL method transforms each datasets correlation coefficient to a Fisher Z value with a standard error determined by the site-specific sample size. Using the sample size allows sites with larger sample sizes for the female fertility outcome to be weighted higher than sites with lower sample sizes. A summary correlation coefficient was computed and represents the overall correlation obtained from the meta-analysis across all sites included from the retrieved studies. We used the DSL method incorporated in the R ‘metacor’ library^25^ with widespread use among the research community^26^ and implemented based on Schulze method^27^.

Humidity is another important factor in the female fertility – birth season relationship^28,29^. However, data on humidity was not available across all included sites and therefore rainfall was used instead. We also investigated the relationship between rainfall as measured in millimetres (mm) per month and female fertility outcomes. The same meta-analysis method was used (DSL) to investigate the relationship between rainfall and female fertility.

## Results

### Systematic review

**Figure 1:**
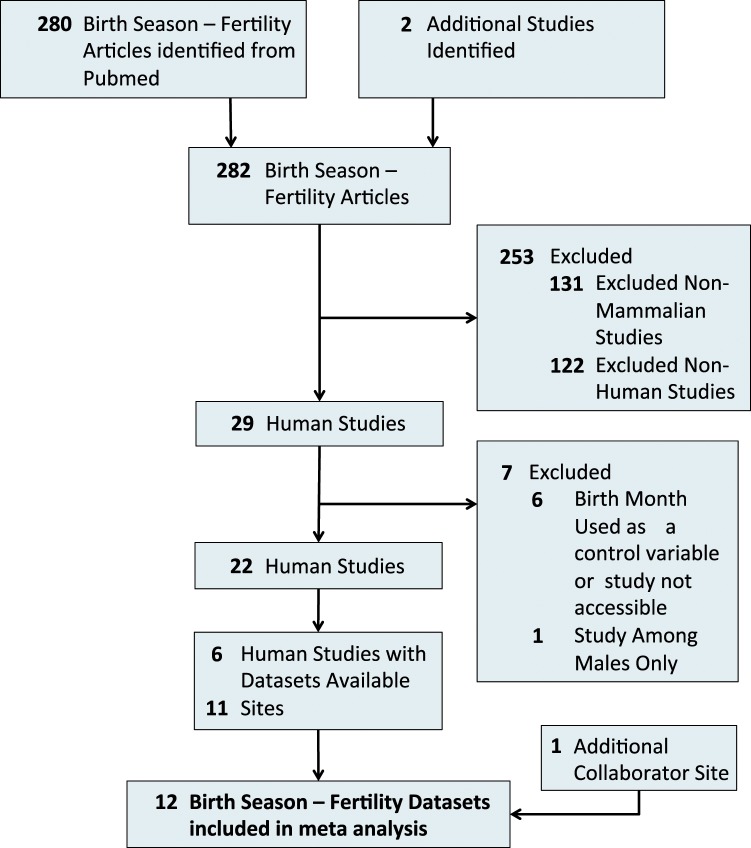
Diagram of the Study Selection Process for the Systematic Review and Meta-analysis According to PRISMA.

Our method for selecting and including studies in our review is shown in Fig. 1 according to PRISMA guidelines^22^. This resulted in an initial set of 280 papers. We added two birth season studies that we know to have fertility-related birth month data, but were from studies exploring multiple birth season outcomes in general and not explicitly ‘fertility’ and ‘reproductive’ studies^10,30^. Our final dataset contained 282 studies. Within these 282 reviewed studies, 122 were non-human mammalian studies that we excluded. In addition, 131 studies were investigating birth season affects in either non-mammalian species (e.g., marsupials, reptiles, fish) or plant species (N = 49 studies). We excluded these from further analysis because the underlying biological mechanisms underlying the role of a birth season mechanism on fertility were morphologically distinct. In total 29 studies were conducted in humans, we further reviewed these for inclusion in our meta-analysis.

Six studies were excluded from the 29 human studies because either a.) birth month was used as a control variable or b.) it was not possible to access the text. Of the remaining 23 relevant human studies, one was about the birth seasonality of males and its effects on reproductive performance. While the focus of this study is on female reproductive performance and birth season, we discuss male studies in section 4.10 because male factor infertility contributes to the total number of offspring born to a woman. However, a sub-analysis of male studies was not possible because of the paucity of studies on male birth season and fertility later in life.

### Meta-Analysis results of female fertility and temperature

Of the 29 human studies, 6 were not relevant because either a.) birth month was used as a control variable or b.) it was not possible to access the full text, resulting in 23 human studies with accessible data. Of these 23 relevant human studies, one was about the birth seasonality of males and its effects on reproductive performance while the remaining 22 studies were on females. We were focusing our meta-analysis on the relationship between female birth month and later fertility. We carefully reviewed each of these 22 studies and included all studies reporting data at the 12-month level that were investigating the effect of maternal birth month on either: a.) total number of offspring or b.) risk of having a child (i.e., giving birth or delivering). From the 22 studies, we were able to extract data on female birth month (in 12-month increments) and later fertility from 6 distinct studies. These 6 studies contained 11 separate datasets. To augment our analyses, we included an additional dataset from our collaborators (co-authors LHJ and PRJ). In total, our meta-analysis consisted of twelve datasets for use in investigating the role of maternal birth season, temperature and rainfall.

Four datasets contain sites from the Asia and the Pacific, four datasets contain sites from Europe and four datasets contain data from the United States of America. Data from 5 sites were reported as the relative risk (RR) of birth given the population (Table 1). For purposes of our meta-analysis, the sample size of women giving birth (i.e., the case sample size) was included for those 5 sites reporting the RR by maternal birth month. The overall population size is also given in Table 1 because those not giving birth were used as controls. This also normalizes for the background birth rate by birth month found at different locations. In the literature, the main reported outcome thought to explain the relationship between female fertility and birth season is temperature. We used the DSL method to compute an overall correlation between temperature and female fertility in humans (R = 0.230, p = 0.135). The individual site-specific correlations and p-values are given in Table 2.

**Table 1:** Female Fertility and Birth Season Studies In Humans Included in Meta-Analysis (N = 6 Studies and 12 Datasets).

Location	Number with Delivery	Total Population	Age (median)	% White	Original Study Reported Findings	Ref.
**United States of America**						
Columbia University Medical Center, New York City, NY, USA	60,584	1,749,400	38	38%	Inversely Correlated with Temperature	^10^
Mount Sinai Hospital, New York City, NY, USA	9,136	1,169,599	53	36%	Female Fertility Outcomes Not Discussed	^30^
University of Washington, Seattle, WA, USA	8,458	1,770,510	48	56%	Female Fertility Outcomes Not Discussed	^30^
Vanderbilt University, Nashville, TN, USA	9,472	3,051,997	44	54%	Female Fertility Outcomes Not Discussed	^30^
**Europe**						
Rotterdam, The Netherlands	32,336	2,018,869	47	NA		From Collaborators
Austria	2,839				Inversely Correlated with Temperature	^8^
Romania, Low Education	294,026		NA	NA	Positively Correlated with Temperature	^34^
Romania, High Education	116,858		NA	NA	Trend towards positive correlation with temperature	^34^
**Asia**						
North Vietnam	196,752		NA	NA	Positively Correlated with Temperature	^33^
South Vietnam	181,835		NA	NA	Positively Correlated with Temperature	^33^
Central Vietnam	115,266		NA	NA	Positively Correlated with Temperature	^33^
**Australasia**						
New Zealand	50,000*		NA	NA	Inversely Correlated with Temperature	^32^

*Sampling with Replacement.

**Table 2:** Results of Relationship Between Female Fertility and Both Temperature and Rainfall at Birth. (N = 6 Studies and 12 Datasets).

Location	Sample Size with Delivery	Total Population	Temperature Correlation, P-value	Rainfall Correlation, P-value	Reference
**United States of America**					
Columbia University Medical Center, New York City, NY, USA	60,584	1,749,400	−0.5, p = 0.1	−0.3, p = 0.3	^10^
Mount Sinai Hospital, New York City, NY, USA	9,136	1,169,599	−0.4, p = 0.2	0.4, p = 0.4	^30^
University of Washington, Seattle, WA, USA	8,458	1,770,510	−0.4, p = 0.1	0.5, p = 0.1	^30^
Vanderbilt University, Nashville, TN, USA	9,472	3,051,997	−0.2, p = 0.6	−0.1, p = 0.7	^30^
**Europe**					
Rotterdam, The Netherlands	32,336	2,018,869	0.9, p < 0.05	0.3, p = 0.4	From Collaborators
Austria	2,839		−0.7, p < 0.05	−0.7, p < 0.05	^8^
Romania, Low Education	294,026		0.9, p < 0.05	0.8, p < 0.05	^34^
Romania, High Education	116,858		0.3, p = 0.3	0.2, p = 0.5	^34^
**Asia**					
North Vietnam	196,752		0.7, p < 0.05	0.7, p < 0.05	^33^
South Vietnam	181,835		0.8, p < 0.05	0.2, p = 0.5	^33^
Central Vietnam	115,266		0.7, p < 0.05	−0.5, p = 0.1	^33^
**Australasia**					
New Zealand	50,000 *		−0.7, p < 0.05	0.7, p < 0.05	^32^

**Figure 2:**
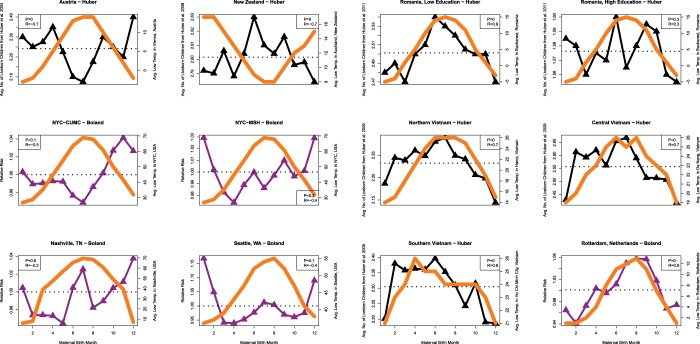
Relationship between Perinatal Temperature and Female Fertility Outcomes. The seasonality of temperature is shown in dark orange while the birth season – fertility relationship is shown in black (Huber *et al*. studies) or purple (Boland *et al*. studies).

Figure 2 shows the relationship between temperature and female fertility outcomes. The seasonality of temperature is shown in dark orange while the birth season – fertility relationship is shown in black (Huber *et al*. studies) or purple (Boland *et al*. studies). Six studies demonstrate positive correlation between temperature and female fertility with perinatal increases in temperature increasing the woman’s fertility later in life (six datasets on the right-hand side of Fig. 2). On the other hand, six studies demonstrated negative correlation between temperature and female fertility with perinatal increases in temperature *reducing* the woman’s fertility later in life (six datasets on the left-hand side of Fig. 2).

### Meta-Analysis results of female fertility and rainfall

We also investigated the relationship between rainfall and female fertility because humidity was another important factor in the female fertility – birth season relationship^28,29^. Because humidity was not directly available, we used the average rainfall in millimetres (mm). Depending on the climate for a given region, some regions are very moist (i.e., high humidity and/or rainfall) and cold while other climates are moist and hot^31^. Similarly, there are climates that are dry and hot and others that are dry and cold. Therefore, the relationship between temperature and humidity depends on the climate of the region, and are not necessarily directly correlated^31^.

**Figure 3:**
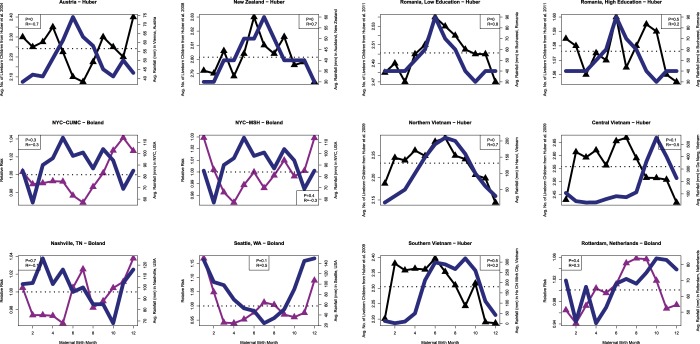
Relationship between Perinatal Monthly Rainfall (mm) and Female Fertility Outcomes. The seasonality of rainfall is shown in dark blue while the birth season – fertility relationship is shown in black (Huber *et al*. studies) or purple (Boland *et al*. studies).

**Figure 4:**
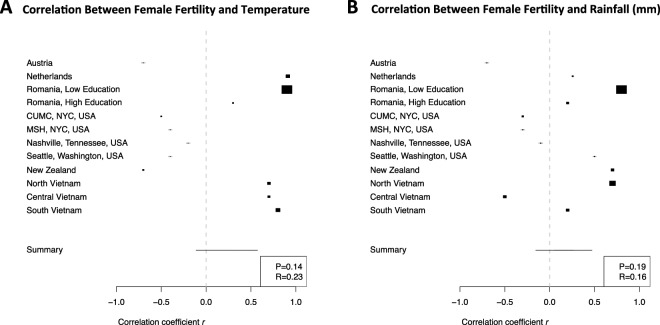
Correlation between Perinatal Temperature (**A**) and Rainfall (**B**) with Female Fertility Outcomes Across All 12 Datasets. Four datasets contain sites from the Asia and the Pacific, four datasets contain sites from Europe and four datasets contain data from the United States of America. Larger squares in Fig. 4 denote correlations with larger confidence intervals while those with smaller squares represent tighter correlations. Therefore, smaller squares typically denote sites where the sample size was large.

Figure 3 shows the relationship between rainfall and fertility at the dataset level. Romania, New Zealand and Seattle, Washington all demonstrated positive correlations between increases in rainfall and *increases* in female fertility. The overall correlation was not significant with an R = 0.158, p = 0.187. The meta-analysis results for temperature are given in Fig. 4A and for rainfall are given in Fig. 4B. These results together appear to suggest the possibility of a combined mechanism between temperature and rainfall where rainfall only *increases* fertility in higher altitude locations (New Zealand, Romania, and Northern Vietnam).

### Quality assessment of included studies

Five of the included studies shown in Fig. 2 and Fig. 3 were the result of studies by Boland *et al*.^10,30^ those studies used data obtained from Electronic Health Records regarding conditions diagnosed among women who are delivering babies. The birth months of these women were assessed and the curves presented in Figs. 2 and 3 represent the ‘relative risk’ of having a baby by birth month. The other 7 studies included in this meta-analysis were the result of Huber *et al*.^8,32–35^. In the Huber *et al*. studies the results are the average number of live-born children per woman by birth month. The Huber *et al*. studies followed women longitudinally over time and therefore it is likely they have a greater capture over a woman’s lifetime reproductive success (i.e., total number of children born to a given woman). Therefore, these two sets of studies differ somewhat in their data collection methods and are not completely comparable. However, it is important to note that findings from the NYC CUMC dataset from Boland *et al*. correlated well with Austria’s data from the Huber *et al*. study indicating that while the studies use different methods the results appear similar.

A major issue recognized by all of the authors of these studies is the lack of adequate confounder adjustment with regards to socioeconomic status and a woman’s choice regarding her desire to have a child (or the lack thereof). The Huber *et al*. Romanian study tried to address this issue by separating out the low vs. high education women as a proxy for socioeconomic status. However, education as a proxy for socioeconomic status is somewhat limiting. The studies using the Electronic Health Records did not have adequate socioeconomic confounder adjustment and therefore this represents a significant confounder in these analyses. Other limitations are discussed in the limitations section.

## Discussion

Our comprehensive review of the literature on the birth season relationship among women with later reproductive performance has revealed that temperature and rainfall are not enough to explain the relationship observed in multiple sites throughout the entire world. However, this review does reveal that there appears to be strong support for a relationship between temperature and reproductive success, including conception rates in bovines^36^ and humans^37^. Some studies also point to the role of the photoperiod over temperature itself as the main driver behind conception rate increases^38^. However, conception rates are notoriously difficult to compare. Studies have shown that the probability of an offspring being born in certain birth months also depends on the birth month of the mother^39^. The authors demonstrated that while May – July born babies were uncommon in general, women who were already born in May – July were more likely to give birth to their offspring during the same months^39^. It is difficult to tease out some of these relationships because foetal loss and conception is generally difficult to capture.

### Principal findings

Our meta-analysis of 12 datasets from around the world has revealed the there is a birth seasonal mechanism underlying female fertility. In addition, it does appear to have a negative correlation with temperature in some regions (Fig. 2). However, the overall meta-analysis statistic revealed a non-significant positive correlation (R = 0.230, p = 0.135) between increasing temperature at birth and increased fertility later in life. We also investigated the relationship between rainfall at the time of birth and later female fertility and found that at the meta-analysis level there was no relationship (R = 0.158, p = 0.187), however certain sites did show strong positive correlation between increased rainfall at birth and later fertility (Fig. 3). In particular, New Zealand, Romania, Northern Vietnam and Seattle Washington appeared to demonstrate correlations between rainfall and female fertility. Each of these regions is mountainous, which could suggest a combined relationship between altitude, and rainfall. Overall, we did not find a single climate variable that could explain the birth seasonality of female fertility across all of the datasets indicating that the relationship between female fertility and birth season is highly complex. The remaining of our discussion focuses on birth season mechanisms that affect female fertility as described in the literature.

### Assessing the etiologies of birth season - female fertility associations

Birth season has been linked with female fertility in multiple studies. In addition, while not statistically significant, there appears to be correlation in certain sites between a.) increased temperature at birth and later reductions in fertility and b.) increased rainfall at birth and increased fertility later in life. Therefore, we synthesize the literature on the birth season – female fertility relationship to explore other factors that modulate this relationship that have not been investigated in current human fertility-birth season studies.

### Differences between seasonal conception rate and maternal birth month

The seasonality of female fertility varies by region. A Tanzanian study in humans found that conceptions were highest among the hot months when compared to the rainy season (Tanzania only has 2-seasons)^37^. Others have found seasonal changes in environmental light intensity preceded changes in conception rates in both Germany and the Netherlands^38^. These findings point to the photoperiod being the primary driver underlying differences in conception rates across the seasons^40^. These studies were investigating *conception rate* and how it varies seasonally. However, our primary interest is the relationship between *maternal birth season* and later fertility outcomes. Perhaps women born in less favoured seasons (i.e., birth seasons corresponding to a less-favoured conception season) are biological different then those from favoured seasons (i.e., birth seasons corresponding to a successful conception season). Some research suggests that successful conceptions that occur in less biologically favoured time periods (i.e., conceiving in a low conception rate month) may have different characteristics, which could possibly explain some birth season effects^39^.

### Timing of menarche and menopause depends on birth season

Other fertility-related processes have been linked to birth season including the time of menarche^41^ and menopause^42^ in women. A study conducted in China consisting of 132,373 females with natural menopause investigated the relationship between birth season and both age at menopause (i.e., cessation of monthly menses) and reproductive timespan^43^. In addition, they had 285,186 females with timing of menarche (i.e., first monthly menses) to investigate any relationship between menarche and birth season^43^. They found that timing of both menarche and menopause was birth season dependent with those born in the spring months (March, April and May) having early menarche and early menopause^43^. This would likely indicate that these women had shorter reproductive timespans. Our meta-analysis did not include the timing of menarche or menopause because these data were not collected for the datasets included in our study. This is a factor to consider in future birth season studies.

### Maternal birth season determines timing of marriage, schooling and fertility effects

The completed fertility of a given woman (i.e., the total number of children born to a woman at the end of her reproductive years) is affected by a number of sociological factors, including a woman’s choice or desire for children. Furthermore, timing of school and age relative to one’s peers has been shown to affect timing of marriage^44^, which affected the age of the woman at the time of her first childbirth. Therefore, studies that investigate the relationship between maternal birth month and probability of having one child will likely show a birth month relationship. This is because there is a relationship between the first-born child and maternal birth month but not completed fertility rate. The completed fertility rate is the total number of children born to a woman at the time when she enters menopause (i.e., she is no longer fertile). The completed fertility rate was not affected by birth month indicating that women can ‘catch up’ later in life by having more children in their later years^44^. Some studies included in our meta-analysis consisted of the total number of offspring born per woman, which is believed to be the completed fertility rate (i.e., Huber *et al*. studies in Figs. 2 and 3). However, other studies included in the meta-analysis investigate the risk of having a child per maternal birth month (i.e., Boland *et al*.), which may be affected by the timing of marriage (this information was not available to the study authors). Importantly, birth season associations were found in the Huber *et al*. studies for completed fertility, which appears to contradict the findings from this Swedish study^44^.

The Swedish study found that women tended to have their first child on average 5.5 years after completing their highest level of education^44^. For example, if a woman were to complete her Medical Degree (MD) at an age of 26 years (which is the custom in the United States of America), the data from Sweden would suggest that this woman would give birth to her first child at 31.5 years of age. On the other hand, a woman graduating with a high-school degree, at 18 years, with no intentions of completing any additional schooling would then be expected to have her first child at approximately 23.5 years of age. The age of a woman at the time of her first childbirth would be expected to affect her total fertility rate with those having their first child at a younger age having more children over their lifetime. However, this is not what the researchers found. They found that women having children at older ages were able to ‘catch up’ and had similar family sizes to those who gave birth at a younger age. However, it is possible that in some countries and areas of the world, it may not be possible for women to ‘catch up’ and therefore, birth season effects may still be observed in those regions.

Importantly, the 7 Huber *et al*. datasets included in our meta-analysis looked at completed fertility (i.e., the total number of offspring born to a woman at the completion of her fertility period) while the 5 Boland *et al*. datasets investigated the relative risk (RR) of having a child. The RR of having a child includes those having their first child and also those having subsequent children. Therefore, the timing of marriage and the likelihood of advanced degrees would be expected to affect the Boland *et al*. datasets (highlighted in purple in Fig. 2 and Fig. 3). However, in general there is agreement between Huber *et al*. and Boland *et al*. studies, and the differences appear to be more driven by the location and the climate characteristics at those locations. Importantly, one study was able to appropriately model martial duration, literacy, nativity, urbanity and birth cohort still found a significant birth season effect in female reproductive performance^45^.

### Maternal birth season determines characteristics of offspring

Several studies have investigated the characteristics of the offspring and how this pertains to the *maternal* birth season. One study, using data from Canada, found that maternal birth season affected the sex ratio of the woman’s offspring^46^. Women born in February through April had a lower sex ratio among their offspring than other maternal birth months^46^. However, the father’s season of birth did not affect the sex ratio of the offspring suggesting that the effect is related to a maternal birth seasonal mechanism^46^. Others have shown that there is seasonality in the sex ratio of children born in Russia however, the authors did not investigate the relationship between maternal birth season and sex ratio^47^.

A historical study using French-Canadian data from 1621 through 1765 found that women born in May through July were more likely to conceive quickly following marriage^48^. In addition, women born between May – July were more likely to offspring in a random birth month distribution (i.e., not skewed towards certain birth months)^48^. A study in the early twentieth century in Japan, investigating records from 1925 through 1960, found that the least common time to be born was May – July^49^. However, women that were born between May and July had offspring that were randomly distributed across the birth months, this result was similar to what was observed in the early French-Canadian dataset^49^. The less season-sensitive women (born between May – July) were more fertile overall^49^, however, there were fewer women born in that period.

The dearth of babies born between May – July could result from selective foetal loss due to some seasonal infection or other seasonal mechanisms^49^. In addition, the authors suggest that perhaps women born between May – July are immune to the infection (or other seasonal agent) that predominates in that season. Their justification for this hypothesis was that while May – July born babies were uncommon in general, women who were already born in May – July were more likely to have an offspring also born during the same birth period^39^. Suggesting that women born in that period may be immune to the factors causing spontaneous foetal loss among women born during other birth months^39^.

A separate study with seemingly concordant results conducted in Spain found that large families tended to have less variability among birth months then smaller families^50^. This indicates the possibility of some type of family-based birth month selection among larger families^50^. Taken together with the prior studies conducted in French-Canada and Japan, there is the possibility that women born in fertile months (e.g., May through July) are having more offspring in general (and having larger families) and are also more likely to carry a baby due in May through July to term (i.e., lower rates of foetal loss among May – July offspring born to May – July mothers). All of these factors could manifest in a preferential family-based selection of birth months among offspring born in large families, as reported in the Spanish study^50^. However, the lower rates of foetal loss among May – July offspring born to May – July mothers indicates that there are birth season affects on the offspring that are due both to the child’s birth month and also its’ mothers birth month.

Other birth season-dependent affects that modulate the mother – offspring relationship include studies conducted on blood type. Seasonal variation among blood types was observed for all ABO blood groups^51^. The ratio of blood type O over A (O/A ratio) was lower among O mothers then A mothers among those born during August-January^51^. Typically, O mothers would be expected to have a higher O/A ratio among their offspring then blood type A mothers. Therefore, having a lower ratio among those born during August through January indicates the possibility of some underlying seasonally dependent infection or immunological response^51^.

### Seasonality of lactation and breast feeding

An Israeli study of Bedouin Arabs found that infants born between January and May were more likely to still be exclusively breastfed (i.e., no supplementation with bottle feeding) at 4-months old than those born between July-October with a difference of 29.4% vs. 19%^52^. The likelihood of a primiparous woman to breastfeed also depended on the birth season of her offspring^53^. They also found that women giving birth in the spring-summer months were more likely to report milk insufficiency as the reason for introducing bottle feeding^54^. Furthermore, being breastfed for a longer period of time, which appears to be seasonal, has been correlated with a number of beneficial outcomes later in life, including lower burden of respiratory illnesses^55^. Therefore, the seasonality of female fertility and birth season could be related to whether or not the female was breastfed as an infant.

### Seasonality of success from assistive reproductive technologies

Studies have shown that *in vitro fertilization (IVF)* success rates depend on season of implantation^56^. These findings have led some to suggest that season of conception should be considered for women who are considering using Assistive Reproductive Technologies (ART) in general^57^. Furthermore, many studies investigating the outcomes of ART now use birth season and year to match cases and controls, indicating the importance of these variables in the outcomes^58^. The highest fertilization rates and also quality-A embryo rates were observed during the spring with the lowest rates being observed during autumn in humans^56^.

### Birth seasonality of circulating hormones

Female birth season also affected circulating serum Estradiol levels^59^. Women born in the spring and summer months had higher levels of Estradiol on the 22^nd^ day of their cycle and higher levels of Sex Hormone-Binding Globulin (SHBG) on both the 11^th^ and 22^nd^ days of their cycle^59^. Physical activity between 13–15 years of age was inversely related to Estradiol levels, even after controlling for age, cycle length, BMI and birth month^59^.

Circulating hormone levels are important for understanding the role of birth season in increasing female fertility. Higher levels of circulating Estradiol were found among women born in spring and summer months^59^. In addition, high Estradiol levels increase the desirability of the female^60^ and also the likelihood of conception^61^. Therefore, the high Estradiol levels associated with certain birth months may increase female fertility by decreasing the time to marriage, which can affect the total number of children born to a female^44^.

### Seasonality of male fertility

Among men, birth season is also a significant factor in fertility. One study from Austria found that men born in the autumn months had fewer offspring and a higher risk of remaining childless than men born in spring^35^. This was the only study demonstrating a birth seasonal relationship with fertility among men in humans.

### Interactions between seasonal mechanisms affecting both female and male fertility

Female birth season has been shown to alter Estradiol levels (section 4.8)^59^. In addition, studies in animals show that male birth season affects a plethora of male factors, including sperm motility^62^ with one human study showing that male birth season can affect reproductive performance^35^. There could be interactions between these two mechanisms, resulting in non-random mating between males and females based on their respective hormonal signatures. At this point, there are not any well-controlled studies in humans looking into this facet of birth season effects in depth.

### Seasonal environmental covariates that may affect the fertility - birth season paradigm

#### Longevity

In addition to the role of birth month on female fertility, researchers have also found that birth month conferred a survival advantage among women born in a certain region. A study using historical data from the Saint Lawrence River region in Canada from women born before the year 1750 found that being born in the winter conferred optimal survival prospects if the child was born in the southern part of the river^63^. However, if born on the northern side of the river, the optimal birth season was during the fall^63^. Women who migrated to different sides of the river appeared to lose their survival advantage indicating that the advantage of birth season on survival may be limited to a certain region^63^. Another group showed that longevity depended on birth month in both men and women^64^. The overall longevity of a woman could impact her complete fertility rate, as she may not live long enough to finish having the children she intended to have. The issue of female mortality on overall fertility rates is still discussed in developing countries^65^ and would be critically important to consider in those countries.

#### Air pollution

Air pollution has also been shown to affect fertility-related outcomes, including pre-term birth^66^. A recent systematic review found that several air pollutants can increase female infertility and related conditions^67^. A study in Barcelona, Spain found a statistically significant reduction in fertility rates with increases in traffic-related air pollution and especially coarse air particulates^68^. Air pollution is important to consider because it also varies seasonally^69^. Therefore, since air pollution varies seasonally, there could be adverse consequences for female babies born in months corresponding to peak exposure to air pollution.

#### Water quality

Variation in water quality can be observed seasonally in many countries^70–72^. This seasonal variation in water quality may affect the developing foetuses born during certain birth months more than other birth months depending on when the exposure occurred (e.g., first or second trimester). This is especially true for certain water exposures, such as Arsenic, which are known to affect the risk of certain adverse reproductive outcomes (e.g., birth weight, foetal loss) and vary seasonally^73,74^.

### Food availability and stress

Food availability in humans is often related to reproductive success^75,76^. In addition, food availability is often seasonally dependent. Therefore, this seasonal process could result in birth month affects for offspring born in periods with low food availability. Stress is another major factor affecting female reproductive performance. One human study found that stress (as measured using cortisol) was important in foetal loss rates^77^.

### Limitations of this review

A major limitation of this review is our interrogation of two related databases – namely PubMed and PubMed Central. PubMed Central contains a subset of papers from PubMed that are open access. Our initial query was through PubMed and then we sought to obtain papers that were either open access (i.e., in PubMed Central) or obtainable via site license agreements through the University of Pennsylvania libraries. PRISMA guidelines recommend that at least one database we searched for relevant articles^78^ and we satisfy this criterion, however it is still a limitation of our work given that some manuscripts may not appear on PubMed Central or PubMed. We were able to locate manuscripts from Austria, Sweden, and many other non-USA countries suggesting that our use of PubMed and PubMed Central was not USA-centric. The major reason that we utilized PubMed and PubMed Central is due to cost, many other online databases such as SCOPUS (https://www.elsevier.com/solutions/scopus) have heavy licensing fees associated with their usage.

In this meta-analysis, we investigate the relationship between female fertility and birth season in published and already collected datasets. We discuss in our discussion section many other variables that were not included in our meta-analysis as these data were not readily available to the researchers conducting the prior studies. These factors are also important in the female fertility – birth season relationship, including socioeconomic factors, timing of menarche, timing of menopause, timing of marriage, a woman’s choice regarding her desire for a specific number of children and so forth. We discuss these other related studies here in our discussion because it is likely that these factors also interact with the female fertility – birth season relationship and may reduce our ability to find a statistically significant p-value on a global scale that adequately explains the female fertility-birth season relationship. Future studies should investigate more fully how these factors may modulate the female fertility-birth season relationship.

### Future work

Future steps should involve research in teasing out the various culprit exposures that appear to affect fertility rates, including stress, heat, and air pollution. Also more work should be conducted on the role of rainfall and female fertility, as some regions appear to demonstrate positive correlations. It is possible that the rainfall in certain regions (Fig. 3**)**, especially mountainous regions (New Zealand, Romania, Seattle Washington) could differ from urban areas (e.g., NYC) with respect to either airborne particulates (that are removed from the air via rainfall) or waterborne contaminants, such as arsenic or lead (with rainfall either increasing or decreasing the exposure). Furthermore, work is needed to investigate if inherited genes are tied to environmental factors and whether or not these factors also play a role in lactation and female fertility. More research is warranted to tease out the gene-environment interactions that may be underlying the female fertility – birth season associations seen throughout the world.

## Conclusion

In conclusion, we systematically reviewed the literature on birth season and female reproductive performance. We performed a meta-analysis on the relationship between temperature at birth and female fertility later in life based on birth month using datasets from 12 sites around the world. We found that temperature at birth was strongly negatively correlated with female fertility later in life only *in certain regions*. However, the overall meta-analysis result showed an insignificant positive correlation between increases in temperature at birth and increases in female fertility (overall: R = 0.23, p = 0.13). We also investigated the relationship between rainfall at birth (in millimetres per month) and female fertility. We found that the overall meta-analysis result was again insignificant with a slight positive correlation (overall R = 0.158, p = 0.187). However, we found that rainfall only *increases* fertility in higher altitude locations (New Zealand, Romania, and Northern Vietnam). This suggests the possibility of a combined or multi-factorial mechanism underlying the female fertility – birth season relationship. We provide an overview of several factors that also vary seasonally by birth month that are involved in the birth season – fertility relationship that may alter the effects observed in certain locales. More work is needed to tease out any potential gene-environment interactions that may pertain to female fertility and birth season and to further explore other confounding variables (e.g., timing of menarche, menopause) that are birth season dependent that could modulate the birth season – fertility relationship.
